# Iberian Odonata distribution: data of the BOS Arthropod Collection (University of Oviedo, Spain)

**DOI:** 10.3897/zookeys.306.5289

**Published:** 2013-06-03

**Authors:** Antonio Torralba-Burrial, Francisco J. Ocharan

**Affiliations:** 1 Universidad de Oviedo, Cluster de Energía, Medioambiente y Cambio Climático, Plaza de Riego 4, 33071, Oviedo, Spain; 2 Universidad de Oviedo, Biología Organismos y Sistemas, 33071, Oviedo, Spain

**Keywords:** Odonata, Arthropoda, Iberian Peninsula, Entomological collections, Biodiversity collections, Distribution, datasets, Spain

## Abstract

Odonata are represented from the Iberian Peninsula by 79 species. However, there exists a significant gap in accessible knowledge about these species,especially regarding their distribution. This data paper describes the specimen-based Odonata data of the Arthropod Collection of the Department of Biología de Organismos y Sistemas (BOS), University of Oviedo, Spain. The specimens were mainly collected from the Iberian Peninsula (98.63% of the data records), especially the northern region. The earliest specimen deposited in the collection dates back to 1950, while the 1980’s and 2000’s are the best-represented time periods. Between 1950 and 2009, 16, 604 Odonata specimens were deposited and are documented in the dataset. Approximately 20% of the specimens belong to the families Coenagrionidae and Calopterygidae. Specimens include the holotype and paratypes of the Iberian subspecies *Calopteryx haemorrhoidalis asturica* Ocharan, 1983 and *Sympetrum vulgatum ibericum* Ocharan, 1985. The complete dataset is also provided in Darwin Core Archive format.

## General description

**Figure 1.**
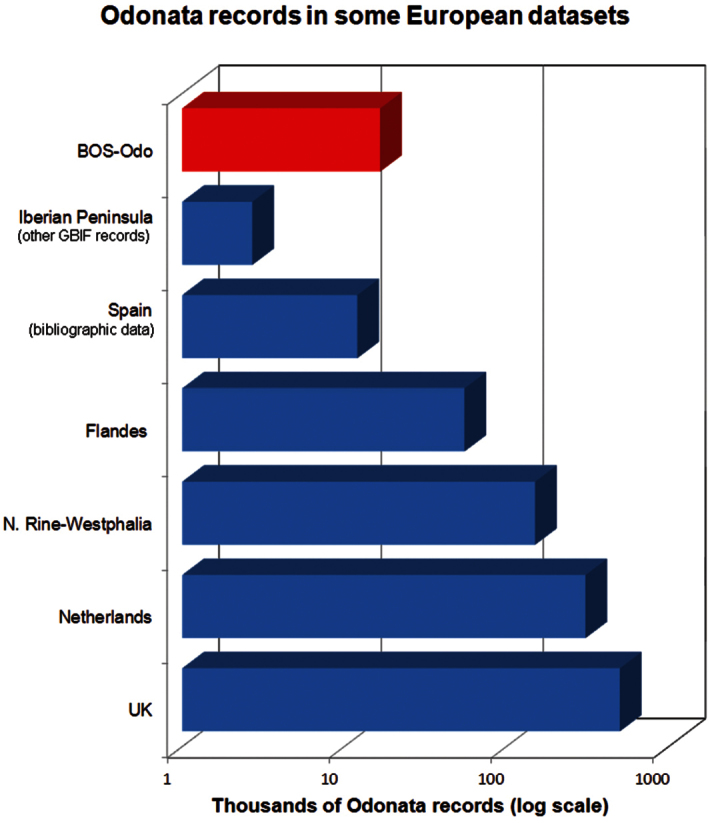
Odonata data records in major European data sets. Sources: BOS-Odo (this dataset); Flandes (De Knijf and Anselin 2010); Iberian Peninsula other GBIF data (GBIF dataportal http://data.gbif.org); Netherlands (Termaat et al. 2010); North Rhine-Westphalia (Conce et al. 2010); United Kingdom (Parr 2010).

**Purpose:** The purpose of this dataset is to make data associated with Odonata specimens deposited in the BOS Arthropod Collection (subcollection of Odonata: BOS-Odo) of the University of Oviedo, Spain. Iberian Odonata (and available data sets) and dragonfly data records are scanty when compared with the distribution data records from other European countries (e.g. Belgium, France, Germany, United Kingdom). Prior to publishing of this dataset, only 2700 data records associated with Iberian Odonata are accessible through GBIF data portal [accessed 2013/04/04], where as nearly 12000 data records from Iberian region for the period 1784-2009 can be tagged or extracted from various publications (includes authors unpublished data), some of which cite the specimens deposited in BOS Arthropod Collection (few listed in reference section). As depicted in Figure 1, other European data sets on dragonfly exceed by far the Iberian available records. For instance, British Odonata database comprises 500,000 records (Parr 2010), Dutch database more than 307,000 (Termaat et al. 2010), North Rhine-Westphalia 150,000 (Conce et al. 2010) or Flandes 55,000 (De Knijf and Anselin 2010). On this backdrop, the BOS-Odo dataset makes significant contribution of primary data about Iberian odonates for ecological, faunistic and conservation studies. Therefore main objective of this data set development were three fold; (1) provide a large dataset with primary distribution data of Iberian Odonata, (2) describe the Odonata subcollection of the BOS Arthopod Collection, (3) promote increasing inhouse and external use of the Collection and the biodiversity data associated.

**Additional information:** A list of publications citing Iberian odonate data contained in this dataset is provided in point 2 of reference section.

## Project details

**Project title:** Informatización de la Colección de Artrópodos BOS de la Universidad de Oviedo / Digitisation of the BOS Arthropod Collection of University of Oviedo.

**Project personnel:** Antonio Torralba-Burrial

**Former curator and promoter:** Francisco J. Ocharan

**Another administrative contact**: Araceli Anadón.

**BOS-Odo collectors**: Collectors who have deposited more than 50 specimens include Antonio Torralba-Burrial, Francisco. J. Ocharan, David Outomuro, Rocío Ocharan, Marta I. Saloña, Antonio Benítez-Donoso, José Alberto Martínez, Saúl Ro-dríguez-Martínez, Matías Brotons-Padilla.

**Funding:** Digitisation of this biological collection is supported by Spanish National R+D+i Plan (MICINN, Spanish Government, grant ref. PTA2010-4108-I) and PCTI Asturias (Asturias Regional Government, ref. COF11-38) through a contract for ATB.

**Study area descriptions/descriptor:** Majority of the Odonata specimens depo-sited in BOS Arthropod Collection are from Iberian Peninsula, which has a geographic extent of 581,300 km^2^, located between latitude 36° and 43°47'N, and between longitude 3°29'E and 9° 29'W, placed at southwest end of Europe. The geographic location and relief distribution of the Iberian Peninsula was responsible for it being glacial refuge (and speciation centre) for many groups of organisms during quaternary period, with limited contact with the rest of the continent. Later on se-veral faunal species belonging to other regions colonised the Iberian Peninsula, which makes it an interesting place for biogeographic or distribution range variations linked to climate change studies. Climatic variation in the Iberian Peninsula is diverse, with annual average air temperature ranges between 2.5 °C in high mountains in the north (Pyrenees) and 17 °C in thermo-Mediterranean zones in the south. Annual average rainfall varies between less than 200 mm in south east (e.g. some zones in Almeria province) and about 2200 mm in the north-west (north Portugal and south Galicia) (AEMET and IM 2011). This climate variation can be analysed in a bioclimatic belts scheme (Rivas-Martínez 1987) or a Köppen-Geiger climate classification system (AEMET and IM 2011). Both systems shown a more humid zone in the north and more dry in the rest of the Iberian Peninsula, although mountain ranges in this zone have their peculiar less dry/less thermic climates.

According to the European Union Habitats Directive (Directive 92/43/CEE), most of the Iberian Peninsula is included in the Mediterranean region, with a narrow band in the north belonging to the Atlantic region and a bit of the Alpine region in the Pyrenees Mountains (biogeographic regions based on vegetation types are the same: Rivas-Martínez et al. 2004). Geographic limits between Mediterranean region and the other are along the southern slopes of the Cantabrian and Pyrenean ranges and in Galicia/northwest of Portugal. Although classification of limnological regions shows the first two bioregions joined in the Iberic-Macaronesian region and the Pyrenees retain as a separate region (Illies 1978, adopted in the Water Framework European Directive, Directive 2000/60/CE) and odonates are aquatic organism, their Iberian distribution seems better explained in the bioclimatic belts scheme (e.g., Brotons et al. 2009, Outomuro et al. 2010).

**Figure 2.**
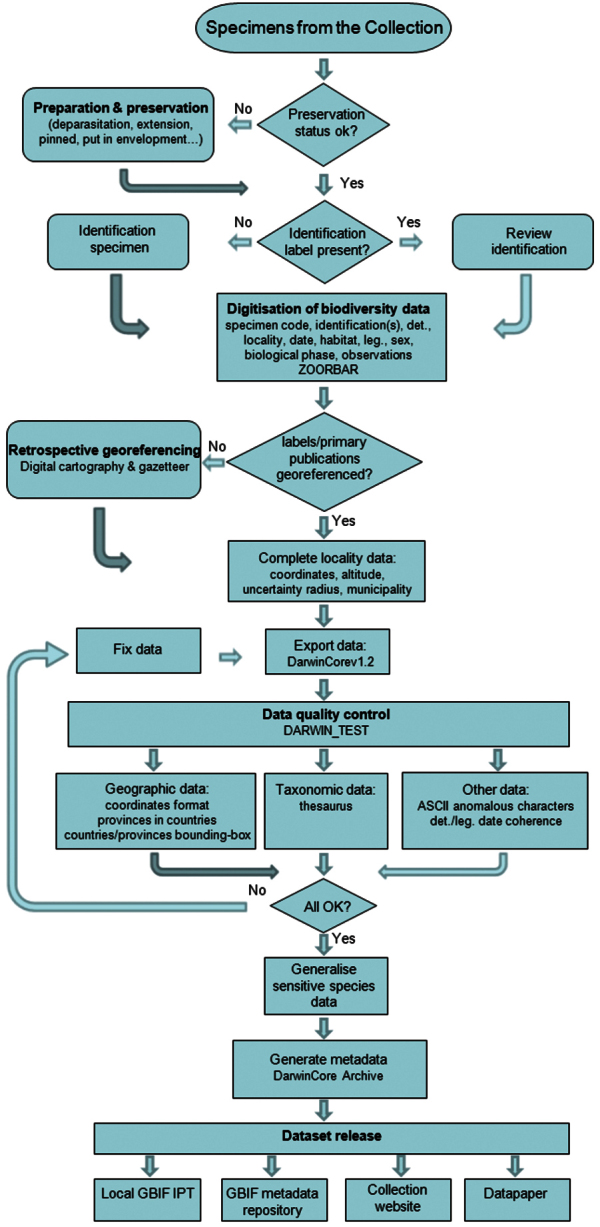
BOS Arthropod Collection digitisation and data publishing workflow.

**Design description:** Figure 2, depicts the digitisation workflow. Prior to digitisation, specimen is carefully examined for its preservation status and if necessary, curative treatment is provided. Subsequent to this taxonomic identification status is examined. In case of non-identified specimens, taxonomic identification is carried out involving experts. Thereafter, data associated with specimens is digitised using ZOORBAR software. In case of absence of precise geo-coordinates, retrospective georeferencing is carried out. If the geo-coordinates are present, they are verified using digital cartography. Best practices as suggested by Chapman 2005a, Chapman and Wieczorek 2006 are followed for the geo-referencing processes. Current accurate spelling of scientific names (Askew 2004, Dijkstra and Lewington 2006) and identification of specimens were reviewed in laboratory with suitable literature (Heidemann and Seidenbusch 2002, Askew 2004, Dijkstra and Lewington 2006, Doucet 2010) as there are explained at quality controls section.

## Taxonomic coverage

**Figure 3.**
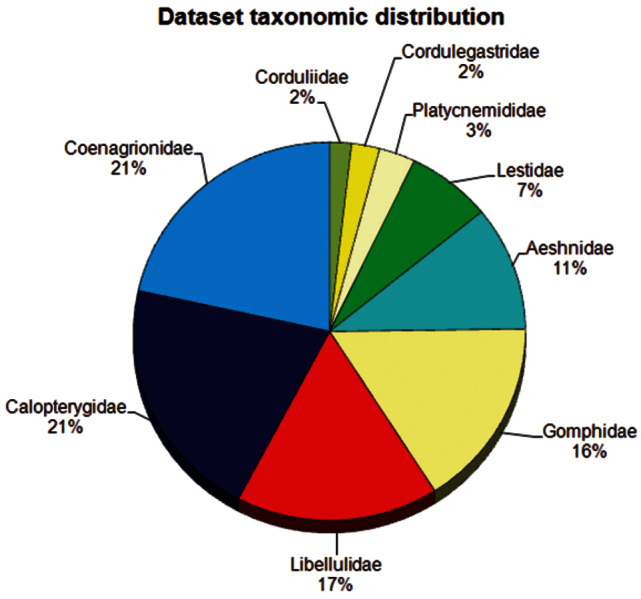
Taxonomic distribution of the Iberian Odonata BOS-Odo dataset.

**General taxonomic coverage description:** All specimens were identified to species or subspecies level with the help of authoritative literature (Heidemann and Seidenbusch 2002, Askew 2004, Dijkstra and Lewington 2006, Doucet 2010) and expert input. All nine dragonfly families recorded from the Iberian Peninsula are present in the collection and dataset. As shown in Figure 3, Coenagrionidae and Calopterygidae are the most abundant families in the BOS Arthropod Collection: each represents approximately 21% of the total specimens deposited. The next most abundant families are the Libellulidae and Gomphidae, each representing approximately 16% of the total specimens deposited. Of the 79 species of known Odonata from the Iberian Peninsula (Torralba Burrial 2009, Mezquita Aranburu et al. 2011), 71 have specimens deposited in the BOS Arthropod Collection. Table 1 provides an account of the number of specimens of key taxa. The BOS Arthropod Collection also includes the holotype and paratypes of the Iberian subspecies *Calopteryx haemorrhoidalis asturica* Ocharan, 1983 and *Sympetrum vulgatum ibericum* Ocharan, 1985 (Table 2). Records of four protected species and nine threatened species (*sensu* the last update of the Spanish Invertebrate Red List: Verdú et al. 2011) are also included in the dataset. Details about the type specimens and those of threatened and protected species housed in the BOS Arthropod Collection are provided in Table 2.

**Table 1.** Taxonomic spread of specimens housed in BOS Arthropod collection.<br/>

**Taxa**	**Nos. of genera**	**Nos. of species**	**Nos. of specimens in the collection**
Family Calopterygidae	1	4	3114
*Genus* *Calopteryx*		4	3114
Family Coenagrionidae	6	14	3578
*Genus* *Ceriagrion*		1	203
*Genus* *Coenagrion*		5	872
*Genus* *Enallagma*		1	434
*Genus* *Erythromma*		2	267
*Genus* *Ischnura*		4	1453
*Genus* *Pyrrhosoma*		1	349
Family Lestidae	3	6	1160
*Genus* *Chalcolestes*		1	403
*Genus* *Lestes*		4	644
*Genus* *Sympecma*		1	113
Family Platycnemididae	1	3	499
*Genus* *Platycnemis*		3	499
Family Aeshnidae		13	1774
*Genus* *Aeshna*		7	481
*Genus* *Anaciaeschna*		1	6
*Genus* *Anax*		3	240
*Genus* *Boyeria*		1	1042
*Genus* *Brachytron*		1	5
Family Cordulegastridae	1	2	387
*Genus* *Cordulegaster*	1	2	387
Family Corduliidae	4	5	297
*Genus* *Cordulia*		1	2
*Genus* *Macromia*		1	2
*Genus* *Oxygastra*		1	290
*Genus* *Somatochlora*		2	3
Family Gomphidae	2	7	2687
*Genus* *Gomphus*		4	97
*Genus* *Onychogomphus*		3	2590
Family Libellulidae	8	24	2778
*Genus* *Brachythemis*		1	13
*Genus* *Crocothemis*		1	321
*Genus* *Leucorrhinia*		1	2
*Genus* *Libellula*		3	179
*Genus* *Orthetrum*		7	565
*Genus* *Selysiothemis*		1	1
*Genus* *Sympetrum*		7	1654
*Genus* *Trithemis*		2	41
*Genus* *Zygonyx*		1	2
TOTAL	27	78	16604

**Table 2.** Type specimens and specimens of threatened and protected species housed in BOS Arthropod collection.<br/>

**Scientific name**	**BOS-Odo ID**	*	**Reference**
*Aeshna juncea* (Linnaeus, 1758)	1201-1213, 4421, 4665-4668, 6736-6737, 9499-9507, 10000-10002, 12120-12126, 13983-13985, 16235-16237	T	Verdú et al. 2011
*Brachytron pratense* (Müller, 1764)	1268-1271, 5668	T	Verdú and Galante 2009
*Calopteryx haemorrhoidalis asturica* Ocharan, 1983	3715	H	Ocharan 1983
	3705-3714, 3716-3756, 3775-3804, 7381-7385,7393	P	Ocharan 1983
*Coenagrion caerulescens* (Fonsbolombe, 1838)	1944-1972, 4096-4100, 4972-4980, 5077-5079, 5777-5079, 5787-5788, 6123-6158, 6560-6561, 7115-7140, 11608, 11830-11831, 14367-14375	T	Verdú et al. 2011
*Coenagrion mercuriale*(Charpentier, 1840)	1877-1943, 3894-4005, 4101-4124, 5109-5125, 5475-5476, 5738-5739, 5759, 6054-6086, 7141-7156, 8102-8106, 8374-8380, 11609-11620, 14354-14362	L	Verdú et al. 2011
*Coenagrion scitulum*(Rambur, 1842)	2012-2035, 4158-4162, 5068-5076, 5766-5770, 8373, 11595-11607, 11810-11819, 14364-14366,	T	Verdú et al. 2011
*Cordulegaster bidentata* Selys, 1843	2847	T	Verdú et al. 2011
*Gomphus graslinii* Rambur, 1842	4655-4656, 7258-7259, 13867-13868, 13883, 13931, 14205-14215, 16072-16074	L	Verdú and Galante 2009
*Gomphus simillimus* Selys, 1840	798, 4561, 5962, 6993, 8048-804, 13869-13882, 13972, 14203-14204, 14401-14402, 15924-15929, 16075-16077, 16128	T	Verdú et al. 2011
*Gomphus vulgatissimus*(Linnaeus, 1758)	797, 5327-5328, 8051-8052, 16241	T	Verdú et al. 2011
*Macromia splendens* (Pictet, 1843)	14197-14198	L	Verdú and Galante 2009
*Onychogomphus costae* Selys, 1885	5963, 6779-6780, 11788	T	Verdú et al. 2011
*Orthetrum nitidinerve*(Selys, 1841)	592-593, 3064	T	Verdú et al. 2011
*Oxygastra curtisii*(Dale, 1834)	789-792, 4333-4335, 8041, 14186-14196, 14269-14275, 14612-14689, 14923-15019, 15543-15596, 16026-16059	L	Verdú and Galante 2009
*Sympetrum flaveolum*(Linnaeus, 1758)	187-193, 2871-2873, 2901, 4633-4634, 4645, 16133-16135, 16209-16233	T	Verdú et al. 2011
*Sympetrum striolatum* (Charpentier, 1840)	16245, 16247	G	Torralba-Burrial and Ocharan 2009
*Sympetrum vulgatum ibericum* Ocharan, 1985	194-206, 5640	P	Ocharan 1985

*: G = gynandromorph, H = holotypus; L = legally protected species; P = paratypus; T = threatened sp. in Spain.

## Taxonomic ranks

**Kingdom:** Animalia

**Phylum:** Arthropoda

**Class:** Insecta

**Order:** Odonata

**Family:** Calopterygidae, Coenagrionidae, Lestidae, Platycnemididae, Aeshnidae, Cordulegastridae, Corduliidae, Gomphidae, Libellulidae.

**Common names:** Dragonflies, Insects, Arthropods.

## Spatial coverage

**Figure 4.**
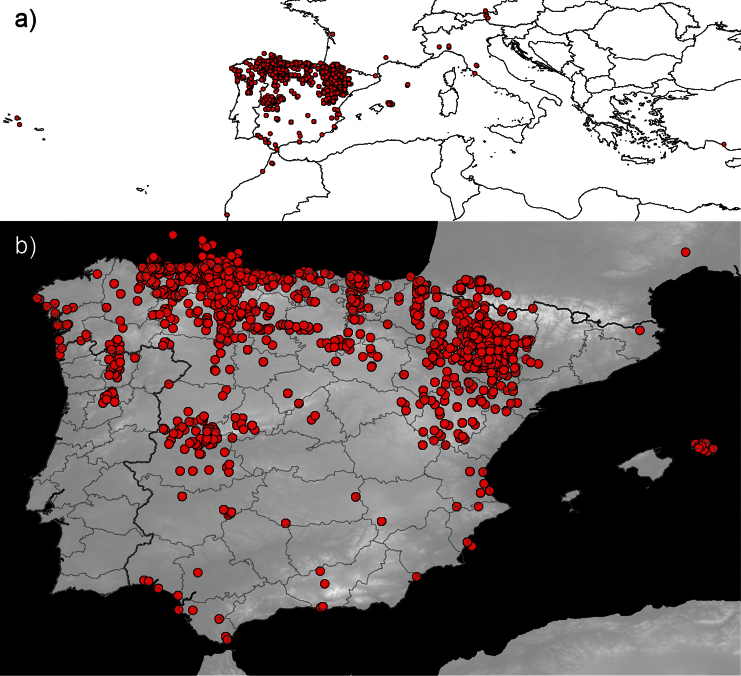
Geographic distribution of specimens in the BOS-Odo dataset: **a** global distribution **b** Iberian distribution.

**Figure 5.**
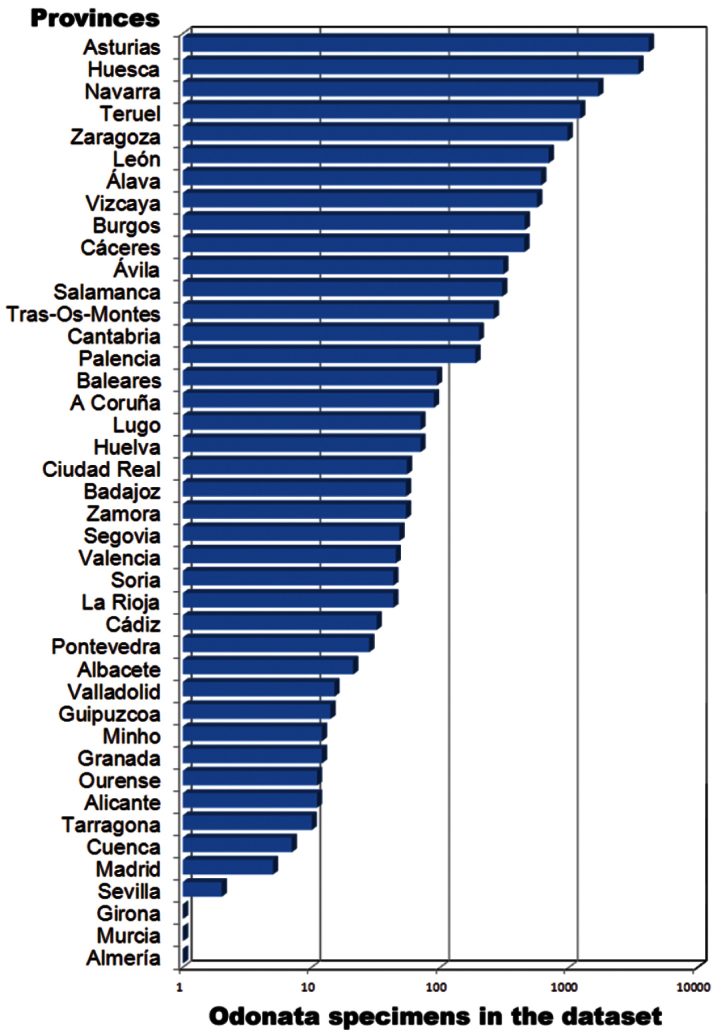
Specimens in each Spanish/Portuguese provinces in the BOS-Odo dataset.

**General spatial coverage:** As evident from Figure 4, majority of the specimens (98.63% of total data) are from the Iberian Peninsula (mainly Spain, but also records of Portugal). The Northern part of the Peninsula is better covered: Asturias (~ 4100 specimens) and Huesca (~3400) are the provinces with more exemplars, followed by Navarra, Teruel, Zaragoza, León, Álava and Vizcaya (between 1700-500 records). Data of other 32 Spanish provinces and 2 old Portuguese continental provinces (3 districts) are available in the database (Fig. 5). Other countries appear in the collection with few specimens: Morocco and Austria, the following countries by specimens, have 40 registers each one.

**Coordinates:** 30°0'0"N and 47°0'0"N Latitude; 27°0'0"W and 32°0'0"E Longitude.

## Temporal coverage

1950 – 2012.

## Natural collections description

**Parent collection identifier:** Colección de Artrópodos BOS

**Collection name:** Colección de Artrópodos BOS de la Universidad de Oviedo: Odonata (BOS-Odo)

**Collection identifier:** http://data.gbif.org/datasets/resource/12776/

**Specimen preservation method:** Specimens are preserved as dry specimens (pinned or in transparent envelopes or in tubes) or in 70° ethanol, sorted alphabetically by family/genus/species and numerically by specimen code in drawers of metallic mobile cabinets in a cold chamber at 6 °C. In drawers with dry specimens paradichlorobenzene is used as insecticide, an additional protection for when the drawers are taken to the lab for study (see Barrientos 2004).

**Curatorial unit:** 16604 with an uncertainty of 0 (Specimens)

## Methods

**Method step description:** The processing workflow is shown in Figure 2. Prior to di-gitisation, odonate specimens in the BOS Arthropod Collection were examined and their preservation status revised: specimens were pinned, labelled and frozen to deparasitize when necessary. The status of the prepared specimens is now as follows: half (48%) of the specimens hosted in the collection are pinned adults, 22% are preserved in 70% ethanol, 18% are dried exuviae and 11% are dry specimens in transparent envelopes. All specimens are stored in metal cabinets in a cold chamber (6°C). More information about these preparation and preservation methods can be found in Entomology handbooks (e.g., Barrientos 2004). Dry specimens pinned before the year 2000 were in entomological boxes with naphthalene as an insecticide; this product was later replaced by paradichlorobenzene. Use of these insecticides does not seem to impede the extraction and amplification of DNA from specimens (Espeland et al. 2010), but it can affect specimens’ colours (Dawson 1988) and researchers’ health (see Guerrero and Corsi 2012 for a recent review of these insecticide effects). The amount of insecticides used in the BOS Arthropod Collection has been reduced in recent years for these reasons.

If a dragonfly specimen had been identified before the digitisation process, then its identification was revised. When the identification label was lacking or incomplete, specimens were identified with suitable literature (see section on quality control). All biodiversity data available on the specimens’ labels (i.e., specimen code, species identification and name of determiner, sex, biological phase, locality, date, habitat, collector and observations) were included in a database using ZOORBAR software (http://www.gbif.es/zoorbar/zoorbar.php), which exports data in Darwin Core (v1.2) format.

A taxonomic thesaurus was developed that includes all synonyms used in Iberian Odonata publications and spelling variants of scientific names. The thesaurus was used to convert the species identifications archived in the offline database (i.e., the species names appearing on the original specimen labels) to the correct/verified scientific name prior to being exported to the online ZOORBAR database.

Other geographic data (municipality, GPS coordinates, altitude, etc.) from specimen labels or associated publications were added to the database when available. GPS coordinates (in UTM/MGRS format) were included without resolution changes (grids of 10 × 10 km or 1 × 1 km are common in entomological studies); ZOORBAR converts the coordinates to decimal degrees and fills out the uncertainty radius at the export data step. Retrospective georeferencing of specimens (see Chapman and Wieczorek 2006) was carried out using digital cartography tools (Google Earth and IBERPIX) if coordinates were not present on the specimen labels or in primary publications. Google Earth can be used to obtain locality coordinates and altitude; it also incorporates a measurement tool that can be used to calculate the uncertainty radius of the place georeferenced. IBERPIX (http://www2.ign.es/iberpix/visoriberpix/visorign.html) is a public gazetteer combining data, maps, satellite images and orthophotographs compiled by the Spanish National Geographic Institute, with a better searchable toponyms database. An accurate, effective, reliable and quick georeferencing process can be acheived by combining the information provided by both tools. Records were sorted geographically for batch retrospective georeferencing, starting with larger batches (Chapman and Wieczorek 2006).

Biodiversity data were exported to a dataset in Darwin Core (v1.2) format. DARWIN_TEST software was used to validate and clean the geographic, taxonomic and additional data associated with the specimens. Erroneous data were corrected and data cleaning was repeated to enhance the data quality (see details in the section on quality control).

Coordinates of threatened species protected by law (e.g., *Macromia splendens*, *Oxygastra curtisii*, *Gomphus graslinii* and *Coenagrion mercuriale*, included in the European Union Habitats Directive and in the Spanish Catalogue of Threatened Species) have been generalised to 0.01° in the online database (see Chapman and Grafton 2008 for details on generalising sensitive data).

The dataset was transformed to a Darwin Core Archive format with metadata and was uploaded to the Integrated Publishing Toolkit (IPT v2.0.4) of the Spanish node of the Global Biodiversity Information Facility (GBIF) (http://www.gbif.es:8080/ipt). On the BOS Arthropod Collection website (http://www.unioviedo.es/BOS/Zoologia/artropodos), links to data pertaining to the BOS odonate specimens included in the GBIF data portal were also provided. The offline version of the dataset includes the identification history of each specimen (17846 items), the habitats in which the specimens were collected, and notes on materials derived from specimens (e.g., microscopic preparations, morphometric data, publications, etc.). This information is available on request.

**Study extent description:** Specimens are mainly from the north half of the Iberian Peninsula, and were collected between 1973 and 2012 (though there are some outliers from other territories or time periods). Half of the Iberian odonate records in the dataset are from the 2000s and a quarter from the 1980s, which can facilitate comparisons over time to assess changes in distribution related to global change, climate change or specific alterations of ecosystems.

**Figure 6.**
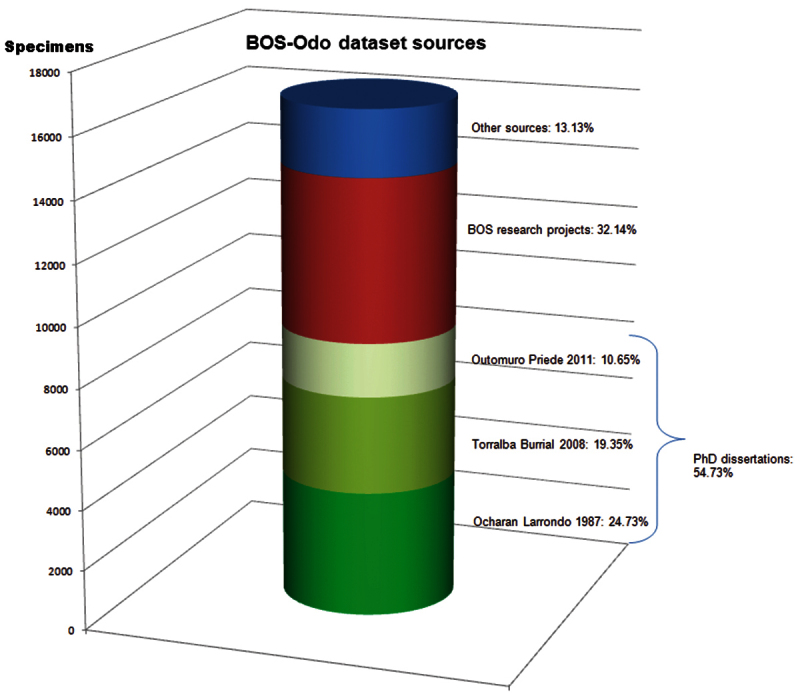
Sources of dragonfly specimens in this dataset.

**Sampling description:** Material deposited in the Odonata subcollection of the BOS Arthropod Collection has been collected in three ways (Fig. 6):

1) Specimens from PhD dissertations carried out at the University of Oviedo (Ocharan Larrondo 1987, Torralba-Burrial 2008, Outomuro Priede 2011) (54.75% of specimens).

2) Specimens collected during research projects, contracts, and development studies in the Department of Biology of Organisms and Systems of the University of Oviedo (32.15%).

3) Specimens from other sources: collections from students in Biology and Forestry Engineering programs at the University of Oviedo, practical courses and other sources (13.1%).

Odonates from source types 1 and 2 were collected using standardized sampling methods according to the requirements of each PhD thesis or project. Specimens from source type 3 are considered to be derived from opportunistic, unplanned and non-standardized sampling. General sampling methods include the collection of adult dragonflies with an entomological net (75.76% of records), exuviae picked directly from a substrate (18.35%), and larvae collected with an entomological net or a Surber sampler (5.82%) (see Barrientos 2004, Torralba-Burrial and Ocharan 2007b).

Specimens deposited by Ocharan (1987) were collected mainly in the provinces of Asturias (42.14%), Guipúzcoa (12.95%), Burgos and Cáceres (both ~8.8%), León (6.57%), Cantabria and Vizcaya (both 3.2%). Sampling was carried out with the intent to capture all species present in each locality. Specimens collected by Torralba-Burrial (2008) are from Aragón, and include adults (184 localities; sampling was performed until all species seen in each locality each day were captured, with a minimum two sampling sessions), larvae (standardized Surber samples from 140 fluvial reaches: Torralba-Burrial and Ocharan 2007a) and exuviae (visual search of the river banks of the 140 reaches sampled, with at least two sampling sessions in each locality). Outomuro’s (2011) specimens are almost all adults of *Calopteryx* spp.

It is not feasible to describe in detail the specific methodologies of each project or study of source 2), but all follow the general guidelines outlined above (examples of different methods in Martínez and Ocharan 2006, Torralba-Burrial and Ocharan 2007b, 2008). In the last few years, conservation efforts have led to a reduction in the collection of adults and an increased use of photographic records (not included in the dataset) unless the sacrifice of specimens is deemed necessary.

**Quality control description:** Validation and cleaning of geographic, taxonomic and additional data associated with the dragonfly specimens was incorporated at se-veral steps of the process (Fig. 2) as an essential component of the digitisation project (see Chapman 2005a, b).

The identifications of all specimens were revised (or performed for the first time when no determination label was available with the original material) by A. Torralba-Burrial between 2010-2012 using suitable literature (adults: Askew 2004, Dijkstra and Lewington 2006; larvae and exuviae: Heidemann and Seidenbusch 2002, Askew 2004, Doucet 2010).

Scientific names on labels were checked with a taxonomic thesaurus. This thesaurus was generated by the authors and includes all synonyms used in Iberian Odonata publications, as well as spelling variants of scientific names. Current European dra-gonfly taxonomy trends (Dijkstra and Lewington 2006, Dijkstra and Kalkman 2012) have been considered in the assignment of valid scientific names. Geographic data appearing on the original specimen labels were cross-checked with known published localities when available. Geographic/UTM/MGRS coordinates shown in published sources were assumed to be correct when no coordinates were included on the labels.

Unique collections’ accession numbers were assigned to each specimen. Other validation procedures, including geographic coordinates format, coordinates within country/provincial boundaries, congruence between collection and identification dates and absence of ASCII anomalous characters in the dataset were checked with DARWIN_TEST (v1.3) software (http://www.gbif.es/darwin_test/Darwin_test.php). Specimens with original MGRS coordinates in a 10 × 10 km grid failed to meet the bounding-box validation in localities near coastlines and country or provincial boundaries, but these coordinates (converted to decimal degrees) were kept in the dataset with the estimated uncertainty radius.

## Datasets

### Dataset description

**Object name:** Darwin Core Archive Iberian Odonata distribution: data of the BOS Arthropod Collection of the University of Oviedo

**Character encoding:** UTF-8

**Format name:** Darwin Core Archive format

**Format version:** 1.0

**Distribution:** http://www.gbif.es:8080/ipt/archive.do?r=Bos-Odo

**Publication date of data:** 2013-04-08

**Language:** Spanish

**Licenses of use:** This dataset [Colección de Artrópodos BOS de la Universidad de Oviedo: Odonata (BOS-Odo)] is made available under the Open Data Commons Attribution License: http://www.opendatacommons.org/licenses/by/1.0/

### External datasets

**Dataset description**

**Object name:** Colección de Artrópodos Biología de Organismos y Sistemas, Oviedo: odonatos

**Character encoding:** iso-8859-1

**Format name:** Darwin Core Archive

**Format version:** 1.0

**Distribution:** http://data.gbif.org/datasets/resource/12776

**Metadata language:** English

**Date of metadata creation:** 2013-03-20

**Hierarchy level:** Dataset
